# Knowledge and vaccination practices among chronic disease patients: Concordance with immunization records -A cross-sectional study

**DOI:** 10.6026/973206300222030

**Published:** 2026-04-30

**Authors:** Kanchan, Aisha Mohammed Kutty, Ujjwala Dehankar3, Usha Sanjay Shende, Sai Vanniappan Meenakshi Sundaram

**Affiliations:** 1 Department of Medicine, University Hospitals Birmingham, West Midlands, United Kingdom; 2 Department of Oncology and Haematology, Portsmouth University Hospital NHS trust, United Kingdom; 3 Department of Microbiology, NKP Salve Institute of Medical Sciences & RC and Lata Mangeshkar Hospital, Nagpur, Maharashtra India; 4 Department of Child Health Nursing, Kasturba Nursing College, Sewagram, Wardha, India; 5 Department of Internal Medicines, Cuddalore Medical College Erstwhile Rajah Muthiah Medical College, Tamil Nadu, India

**Keywords:** Vaccination, chronic disease, immunization practices, patient knowledge, vaccine uptake, questionnaire survey, immunization record review, preventive health behaviour, vaccine adherence, adult immunization

## Abstract

Suboptimal vaccination coverage among individuals with chronic diseases increases the risk of preventable infections and related complications. Therefore, it is of interest to evaluate patient knowledge and vaccination practices and compared self-reported status with documented immunization records among 240 adults with chronic illness. Awareness of recommended vaccines varied widely, with 70% recognizing the COVID-19 booster but only 35% aware of pneumococcal vaccination. Documented coverage for pneumococcal and Tdap vaccines was 20.0% and 13.3%, respectively and discrepancies were observed between self-report and verified records. Thus, higher awareness and provider counselling were associated with improved documented vaccine uptake, highlighting persistent gaps in education and record verification.

## Background:

Vaccination remains a fundamental strategy for preventing infectious diseases and reducing healthcare burden [1]. Individuals with chronic diseases are at increased risk of severe infection, hospitalization and mortality from vaccine-preventable illnesses [2]. Conditions such as diabetes, cardiovascular disease, chronic respiratory disorders and renal impairment alter immune responses and increase susceptibility to complications [3]. National and international guidelines recommend additional adult vaccines for high-risk populations, including influenza, pneumococcal, hepatitis B and Tdap boosters [4]. Despite these recommendations, vaccination coverage among chronically ill adults remains suboptimal worldwide. Studies have identified low awareness, misinformation and inconsistent provider counselling as major contributors to under-immunization [5]. Self-reported vaccination status is frequently used in clinical practice. However, recall bias may lead to inaccurate reporting [6]. Record verification provides a more objective assessment of vaccine uptake and may reveal discrepancies between perceived and documented immunization status [7]. Gaps between knowledge, attitude and verified vaccination coverage highlight missed preventive opportunities [8]. Provider recommendation remains one of the strongest predictors of vaccine acceptance [9]. Limited counselling during routine chronic disease consultations reduces uptake. Educational attainment and health literacy also influence awareness and adherence to immunization schedules [10]. Therefore, it is of interest to evaluate patient knowledge and vaccination practices among individuals with chronic disease and compare self-reported status with documented immunization records.

## Materials and Methods:

A cross-sectional questionnaire-based study was conducted among adults aged 18 years or older with diagnosed chronic diseases attending selected outpatient clinics during the study period. Participants were recruited consecutively if they had lived with a chronic condition for at least six months. Individuals unwilling to provide vaccination records were excluded. A pre-tested structured questionnaire was administered to assess knowledge of recommended adult vaccines, perceived importance of vaccination, prior counselling exposure, beliefs regarding vaccine safety and self-reported immunization status. Sociodemographic and clinical variables were also recorded. Vaccination records were verified through review of vaccination cards, electronic health records or documented clinical files to confirm uptake. Discrepancies between self-reported and documented vaccination status were analysed to evaluate recall accuracy. Data were anonymized and entered into a secured database. Descriptive statistics were used to summarize demographic characteristics, awareness levels and vaccination coverage. Cross-tabulation analysis was performed to examine associations between awareness scores, education level and documented vaccine uptake. Statistical significance was defined as p < 0.05. Institutional ethics approval was obtained and written informed consent was secured prior to participation.

## Results:

A total of 240 adults with chronic diseases were included in the analysis. Most participants were aged between 31 and 60 years, with a slight male predominance. Diabetes and hypertension constituted the most common chronic conditions. Awareness of recommended adult vaccines varied substantially across vaccine types. Knowledge of the COVID-19 booster was highest, while awareness of pneumococcal and Tdap vaccines was considerably lower. Self-reported vaccination uptake was modest across most vaccine categories. Documented verification revealed lower coverage compared to self-reports for several vaccines. Discrepancies were observed between perceived and actual vaccination status. Barriers to vaccination were primarily related to lack of awareness and limited provider recommendation. Higher education level was associated with improved awareness scores. Documented vaccine uptake increased progressively with higher awareness levels. Provider counselling frequency remained suboptimal in a large proportion of participants. Table 1 (see PDF) shows that 67.5% of participants were aged 31-60 years, 53.3% were male and 21.6% had graduate-level education. Table 2 (see PDF) indicates that diabetes mellitus accounted for 42.5% and hypertension for 35.8% of chronic conditions, with 19.2% having multi-morbidity. Table 3 (see PDF) demonstrates that awareness was highest for the COVID-19 booster at 70.0% and lowest for the Tdap booster at 24.2%. Table 4 (see PDF) highlights that self-reported receipt was highest for the COVID-19 booster at 75.8% and lowest for Tdap at 19.2%. Table 5 (see PDF) shows that documented coverage was 70.0% for COVID-19 booster, 40.0% for influenza, 24.2% for hepatitis B, 20.0% for pneumococcal and 13.3% for Tdap. Table 6 (see PDF) indicates that accurate self-report ranged from 72.5% to 88.3%, with over-reporting observed across multiple vaccines. Table 7 (see PDF) demonstrates that lack of awareness was the most frequent barrier at 45.0%, followed by inadequate provider recommendation at 32.5%. Table 8 (see PDF) compares education levels and shows high awareness increasing from 52.9% among those without formal education to 80.8% among graduates. Table 9 (see PDF) depicts that documented vaccine uptake increased from 29.1% in the low-awareness group to 67.5% in the high-awareness group. Table 10 (see PDF) highlights that 40.8% of participants reported never receiving vaccination counselling.

## Discussion:

This study evaluated vaccination knowledge and practices among individuals with chronic disease and compared self-reported status with documented immunization records. The findings reveal substantial gaps in both awareness and verified vaccine uptake. Although attitudes toward vaccination were generally positive, documented coverage remained low for several recommended adult vaccines [11]. Awareness varied considerably across vaccine types. Knowledge of the COVID-19 booster was relatively high, whereas awareness of pneumococcal and Tdap vaccines was limited. These findings suggest selective information exposure. Public health campaigns during the pandemic likely improved COVID-19 awareness, while routine adult immunization education remains insufficient [12]. A significant discrepancy was observed between self-reported and documented vaccination status. Self-reported coverage consistently exceeded record-verified uptake. This pattern indicates recall bias and possible social desirability bias. Reliance on patient recall may therefore overestimate true vaccination coverage. Record verification provides a more objective assessment of immunization gaps [13]. The accuracy of self-report varied by vaccine type. Concordance was highest for COVID-19 booster vaccination. Discrepancies were more pronounced for influenza and hepatitis B vaccines. Differences in recall may reflect vaccine regency or public salience. These findings underscore the need for standardized documentation practices [14]. Lack of awareness emerged as the most common barrier. Nearly half of participants identified insufficient knowledge as a reason for non-vaccination. Inadequate provider recommendation was also frequently reported. Provider endorsement remains a strong determinant of vaccine acceptance. Missed counselling opportunities during chronic disease visits may contribute to low uptake [15]. Educational level was positively associated with awareness. Graduates demonstrated substantially higher awareness compared to individuals without formal education. Health literacy influences understanding of vaccine recommendations and risk perception. However, awareness alone did not ensure universal uptake. Structural and behavioural factors also influenced immunization behaviour [16]. Documented vaccine uptake increased progressively with higher awareness scores. Participants with high awareness were more than twice as likely to have received at least one recommended vaccine compared to those with low awareness. This association indicates that knowledge contributes to preventive action when supported by accessible services [17]. A notable proportion of participants reported never receiving vaccination counselling. Chronic disease consultations often prioritize disease control over preventive care. I ntegration of vaccination review into routine follow-up may reduce missed opportunities. System-level reminders and digital record integration could improve adherence [18]. Multi-morbidity did not correspond with proportionally higher vaccine uptake. Individuals with multiple chronic conditions remain vulnerable yet under-immunized. This finding suggests gaps in targeted preventive strategies for high-risk populations [19]. This study provides region-specific evidence linking awareness levels, education and provider communication with objectively verified immunization coverage. The direct comparison of self-reported and documented vaccination status strengthens the validity of findings. Few studies integrate questionnaire assessment with record verification in chronic disease populations [20].

## Limitations:

Limitations include cross-sectional design and reliance on available documentation. Some records may have been incomplete or fragmented across care settings. However, the discrepancy between recall and documentation remains clinically relevant. Overall, vaccination gaps among individuals with chronic disease reflect deficits in awareness, provider communication and documentation systems. Addressing these determinants is essential for strengthening adult immunization strategies. Accurate record verification and structured counselling should be integrated into chronic disease management frameworks.

## Conclusion:

Vaccination coverage among individuals with chronic disease remains suboptimal, with significant gaps between self-reported and documented immunization status. Limited awareness and inconsistent provider counselling contribute to reduced vaccine uptake. Higher education and greater awareness are associated with improved documented vaccination. Thus, routine verification of immunization records and structured counselling during chronic disease visits are essential to strengthen preventive care delivery.

## References

[1] Johansen ND (2024). JAMA..

[2] Pawar N (2023). Arthritis Care Res (Hoboken)..

[3] Trakarnvanich T (2022). Vaccine..

[4] Ma BM (2021). Nephrology (Carlton)..

[5] Alfaro T (2025). Pulmonology..

[6] Yap DY (2023). Sci Rep..

[7] Proaáos NJ (2023). Int J Chron Obstruct Pulmon Dis..

[8] Vayısoglu Sahin  G (2022). Tuberk Toraks..

[9] Manjappa S (2022). Transplant Cell Ther..

[10] Doneddu PE (2023). Eur J Neurol..

[11] Sanders JF (2022). Transplantation..

[12] Eder M (2021). PLoS One..

[13] Sanders JF (2023). Clin Infect Dis..

[14] Boesing M (2024). Praxis (Bern 1994)..

[15] Fujikura T (2025). Clin Exp Nephrol..

[16] Joean O, Welte T (2022). Expert Rev Respir Med..

[17] Liu C (2022). Zhonghua Jie He He Hu Xi Za Zhi..

[18] Hern án-García C (2025). Front Public Health..

[19] Bhat S (2023). Gastroenterology..

[20] Sui HT (2022). Zhonghua Liu Xing Bing Xue Za Zhi..

